# Biomechanical Effects of Ti-Base Abutment Height on the Dental Implant System: A Finite Element Analysis

**DOI:** 10.3390/jfb15040101

**Published:** 2024-04-11

**Authors:** Miguel Beltrán-Guijarro, Esteban Pérez-Pevida, David Chávarri-Prado, Alejandro Estrada-Martínez, Markel Diéguez-Pereira, Fernando Sánchez-Lasheras, Aritza Brizuela-Velasco

**Affiliations:** 1 Department of Surgery, Faculty of Medicine, University of Salamanca, 37007 Salamanca, Spain; mbeltgui@unizar.es; 2 Department of Surgery, Faculty of Sports and Health Sciences, University of Zaragoza, 22006 Huesca, Spain; 3 Faculty of Health Sciences, Miguel de Cervantes European University, 47012 Valladolid, Spain; davidchavarri@hotmail.com (D.C.-P.); alex.estra12@gmail.com (A.E.-M.); markeldieguez@hotmail.com (M.D.-P.); aritzabrizuela@hotmail.com (A.B.-V.); 4 Department of Mathematics, University Institute of Space Sciences and Technologies of Asturias (ICTEA), University of Oviedo, 33006 Oviedo, Spain; sanchezfernando@uniovi.es

**Keywords:** finite element modelling, dental implant biomechanics, fatigue analysis

## Abstract

This study aims to analyse, using a finite element analysis, the effects of Ti-base abutment height on the distribution and magnitude of transferred load and the resulting bone microstrain in the bone-implant system. A three-dimensional bone model of the mandibular premolar section was created with an implant placed in a juxta-osseous position. Three prosthetic models were designed: a 1 mm-high titanium-base (Ti-base) abutment with an 8 mm-high cemented monolithic zirconia crown was designed for model A, a 2 mm-high Ti-base abutment with a 7 mm-high crown for model B, and a 3 mm-high abutment with a 6 mm-high crown for model C. A static load of 150 N was applied to the central fossa at a six-degree angle with respect to the axial axis of the implant to evaluate the magnitude and distribution of load transfer and microstrain. The results showed a trend towards a direct linear association between the increase in the height of the Ti-base abutments and the increase in the transferred stress and the resulting microstrain to both the prosthetic elements and the bone/implant system. An increase in transferred stress and deformation of all elements of the system, within physiological ranges, was observed as the size of the Ti-base abutment increased.

## 1. Introduction

The digitisation of the dental profession and the development of different materials applicable to computer-aided design and manufacturing (CAD/CAM) workflows in oral rehabilitation represent a major advance in the last decade. The optimisation of the properties of monolithic materials has resulted in the need to develop interfaces for the connection between implants and prostheses. As a consequence, titanium-base (Ti-base) abutments have been developed for the retention of crowns and bridges in monolithic materials, such as zirconium (Zr), either by cement-retained or cement- and screw-retained restorations [1].

Several studies have reported results on the influence of Ti-base abutments on the mechanical–technical complications associated with prostheses. In this regard, the height of abutments could be a factor directly related to the cementation retention capacity of the crowns they support [2].

It should also be noted that the use of intermediate abutments could often be the origin of several biological complications. Several clinical studies assessing the influence of the transgingival height of the abutment on some dependent variables, such as peri-implant marginal bone loss, are of particular interest [3]. The different heights of the Ti-bases result from the need to offer suitable prosthetic solutions that respect the biological spaces typical of implant-borne restorative systems [4].

These mechanical factors with biological influence are described in the literature [5,6]. In 1986, Albrektsson et al. [7] suggested that the success of implant therapy depended on multiple factors, including the importance of the applied load, highlighting the magnification factors of these forces, such as the direction, magnitude and direction of the forces.

It should be considered that the stress transferred to the bone will influence its deformation, and this, in turn, will influence the physiology of the bone and its adaptive response. In 1987, Frost reported that a minimal degree of bone deformation can imply a tendency to resorption due to the absence of load or, on the contrary, deformation can exceed a threshold that tends to produce microfractures due to excess deformation [8].

The increasing use of Ti-base abutments has been evolving, so that the industry provides us with interfaces of different heights, to be able to customise patient treatments as much as possible. The different interface heights are useful for selecting the most suitable one according to factors such as the apical position of the implant or the thickness of the gingival biotype. However, the influence of interface height on the expected biomechanical behaviour is currently unknown.

Technological advances in recent years have provided some tools designed to reproduce biological and mechanical conditions, one of which is finite element analysis (FEA). FEA is a numerical simulation technique widely used in engineering which, through the construction of a theoretical bone-implant-crown model based on a triangulation system, allows the generation of different types of contexts and the analysis of the results. This would enable us, for example, to analyse the biomechanical behaviour of the elements when subjected to a given load, as well as the direction and distribution of this load [9,10].

The model design should simulate the real conditions as accurately as possible. It has to be considered that the union between the implant and the supporting bone is rigid. Since there is no periodontal ligament, it lacks mobility when loads are applied. Therefore, all the stress transferred by the implant will result in the deformation of all the components of the assembly, including the crown, the abutments, and the supporting bone. The FEA has the advantage of being able to modify any element of the system: abutment length, implant size, cortex thickness, etc. Furthermore, it allows the simulation of different load situations and directions of forces, obtaining results for different combinations. It is vital to understand the current status of the use of FEA in dentistry to ensure the validity of the results obtained in our studies [11,12].

Therefore, considering the lack of evidence on the influence of the biomechanical behaviour of Ti-base abutments in relation to their height, a study using FEA is presented to design models with different abutment heights while maintaining the same prosthetic height, with the aim of determining in a theoretical way the ideal abutment height that would allow an optimal bone-implant load transfer in terms of bone stability. The hypothesis is that the prosthetic height of the Ti-base abutments does not influence the amount and distribution of the load transfer to the prosthesis-bone-implant system and the resulting microstrain.

This study aims to analyse the influence of the prosthetic abutment height, in this case Ti-base abutments, on the load transfer to the prosthesis-bone-implant system, and the resulting microstrain of the elements themselves. In turn, the study seeks to transfer these in vitro results to the possible biological consequences in terms of peri-implant marginal bone loss, as well as the mechanical and technical consequences of prosthetic components.

## 2. Materials and Methods

All the elements of the models examined in this study were meshed using regular tetrahedra, which ensures a high convergence of the results. For each model, more than 100,000 tetrahedral elements were generated, with a mesh size an order of magnitude smaller than a millimetre in the critical contact areas.

### 2.1. Finite Element Model Design

A three-dimensional (3D) finite element model was designed to assess the magnitude and distribution of stresses applied to the prosthetic components, implant, and bone, and the resulting microstrain corresponding to the Ti-base abutment of different heights. The bone model produced was an edentulous posterior mandibular section of the premolar area [12,13,14]. A juxta-osseous threaded bone-level implant was modelled and rehabilitated using a 1, 2, or 3 mm Ti-base abutment with a cemented zirconium dioxide (ZrO_2_) crown [10]. Three implant-crown models were therefore developed.

#### 2.1.1. Bone

**Figure 1:**
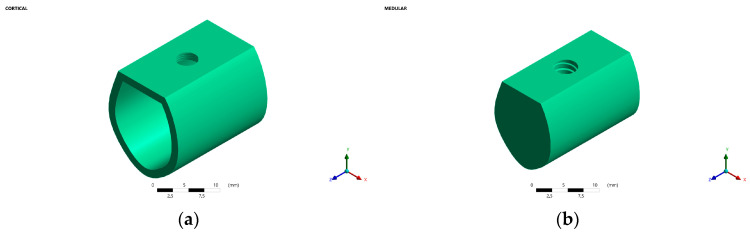
(**a**) Cortical bone 2 mm thick. (**b**) Medullary bone 21 mm high and 10 mm thick.

According to the Leckholm and Zarb classification [15], a type A-2 mandibular section was created, which consisted of a layer of compact cortical bone surrounding a core of dense trabecular bone. This type of bone is the most common for this mandibular region as reported in the literature [13,16,17,18]. The dimensions of the bone section were 23 mm high and 12 mm wide. The cortical bone was 2 mm thick, and it surrounded the rest of the bone section, which had trabecular bone characteristics (Figure 1).

#### 2.1.2. Implant

**Figure 2:**
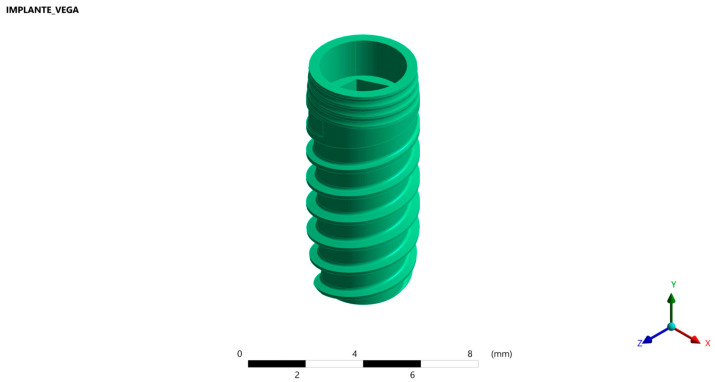
Vega implant system, 4 mm in diameter and 10 mm in length.

To conduct the macroscopic design, a solid threaded bone-level implant with an internal connection and a Ti6Al4V alloy (Vega^®^, Klockner Implant System^®^, Madrid, Spain) [19], whose dimensions were 4 mm in diameter and 10 mm in length, was created. The implant had a conical apex, straight body, a double pitch thread (2.2 mm), and a threaded core that was conical at the apex, straight in the centre of the body, and conical at the most coronal part. The implant neck was convex, cone-shaped and had three microthreads (0.3 mm) with a 0.4 mm gap between them, which allowed the distribution of loads to the adjacent bone tissue, helping to maintain the cortical bone and reducing stress in the crestal region. In the most apical part of its connection, the implant had a hexagonal polygon that facilitated its handling and the correct positioning of the abutment, as well as optimising the precision of the adjustment and minimising rotational movements. The coronal part of the connection consisted of a reverse cone with an angulation of 10° and a length of 1.1 mm that allowed a correct fit between the implant cone and the prosthetic attachment cone, which facilitated the professional insertion and guidance of the different attachments and provided a hermetic seal, less than one micron, that prevented bacterial colonisation inside the implant (Figure 2).

#### 2.1.3. Ti-Base Abutment

Three titanium Ti-base abutments with Ti6Al4V alloy (Klockner Implant System^®^, Madrid, Spain) identical in morphology and connection, but with different transgingival heights of 1, 2 and 3 mm, were modelled.

The height of the selected abutments corresponds to the most clinically used Ti-bases that meet restorative needs [4].

**Figure 3:**
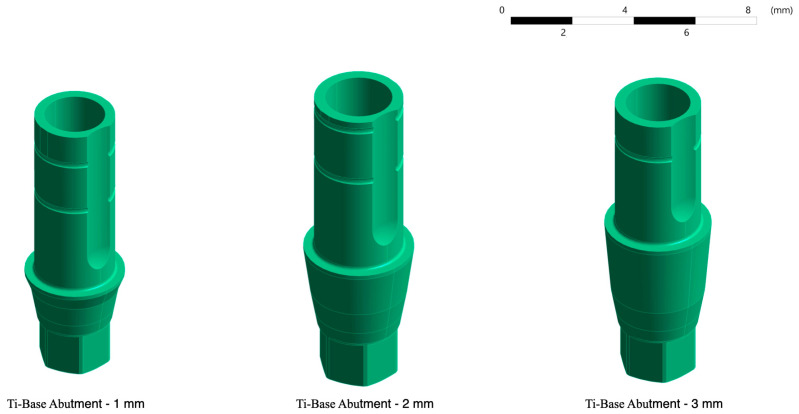
Three-dimensional models of the Ti-base with different transgingival heights.

The design of this abutment allowed it to be screwed into the implant connection once the implant had osseointegrated. In this study, this connection was considered to be complete and effective, regardless of screw preload and other static stresses typical of a screwed system (Figure 3).

#### 2.1.4. Prosthetic Crown

A zirconium dioxide (ZrO_2_) crown was modelled to cement each of the titanium bases. The crown material was selected considering that it is a material commonly used for the fabrication of definitive crowns and bridges.

**Figure 4:**
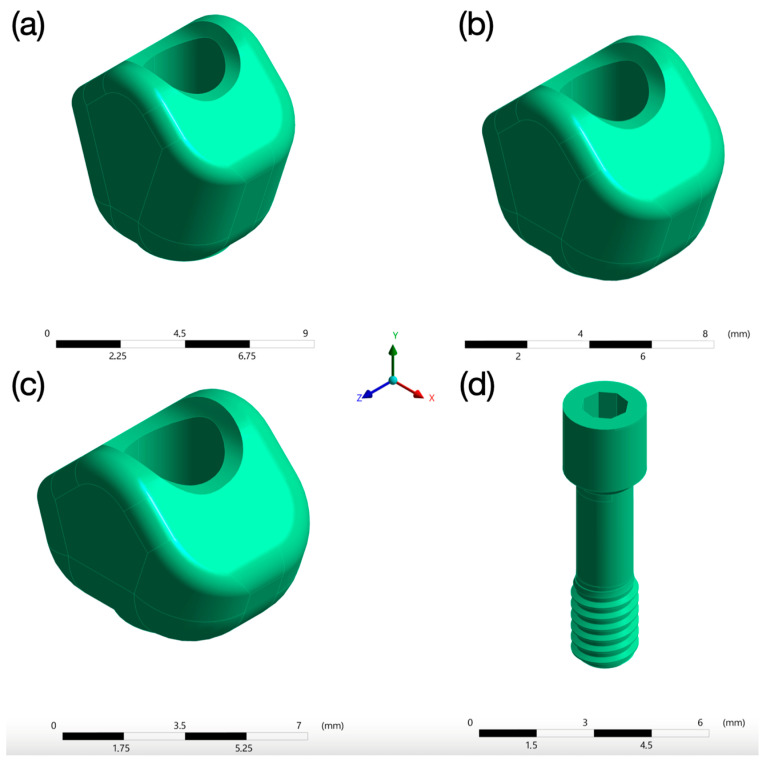
Three-dimensional models of the crowns with 8 mm (**a**), 7 mm (**b**) and 6 mm (**c**) height, respectively, with a thickness of 4 mm, and 3D model of the 7.97 mm long, 2.2 mm diameter prosthetic screw (**d**).

The crowns had heights of 8 mm, 7 mm and 6 mm, and were cemented on Ti-bases of 1, 2 and 3 mm in height, respectively, so that the 9 mm height of the prosthetic assembly was maintained in all models. However, the models did not differ in width (11.5 mm) or thickness (4 mm) (Figure 4a–c).

The junction between the Ti-base and the ZrO_2_ crown was considered tight, firm and effective, regardless of the cementitious medium.

The crowns are attached to the implant using a titanium prosthetic screw (Klockner Implant System^®^, Madrid, Spain) with a length of 7.97 mm and a diameter of 2.2 mm (Figure 4d).

**Figure 5:**
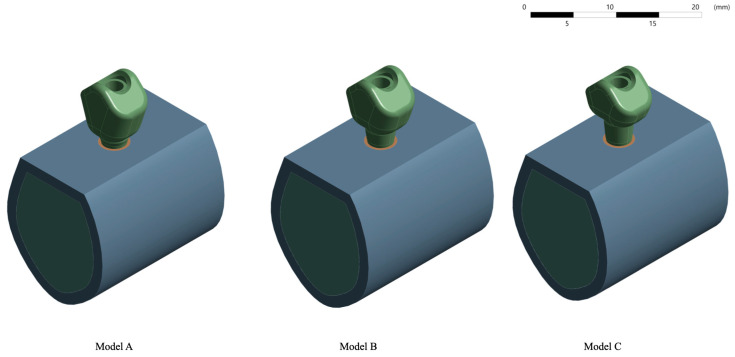
Three-dimensional study models with their corresponding abutments and crowns. (Model **A**) with a 1 mm abutment and an 8 mm crown, (Model **B**) with a 2 mm abutment and a 7 mm crown and (Model **C**) with a 3 mm abutment and a 6 mm crown.

Three models were therefore obtained, model A with a 1 mm abutment, model B with a 2 mm abutment, and model C with a 3 mm abutment (Figure 5).

### 2.2. Material Properties, Interface Conditions, Load and Boundary Conditions

All the components used in this study were computer-modelled through digital design software using the information corresponding to each component, under the assumption that all the materials were homogeneous, isotropic, and had linear elasticity [10].

Furthermore, the attachment of the prosthetic system to the implant was considered complete and effective, regardless of screw preload and other static stresses characteristic of a screw-retained system. Based on assumptions from previous studies, the bond between the bone and the implant as a whole was assumed to be complete, with 100% contact between the implant surface and the bone [20,21,22,23,24]. It is important to note that all the contacts between the parts were securely bonded.

The elastic characteristics given to each of the modelled components were obtained from the literature and expressed in Young’s modulus and Poisson’s ratio and are shown in Table 1 [25,26,27,28].

A computer with eight 16 GB DDR4 modules and a 2 Xeon processor E2690 v3 was used to digitise all the data. The design of the models was performed using Ansys 11.0 (Ansys, Swanson Analysis System, Canonsburg, PA, USA).

The nodes and elements used to create the different models are shown in the following Table 2.

Based on the literature, static loads were used in this study and, since the occlusal force system is non-coplanar and non-concurrent, it can only be reduced to an equivalent force-couple system at any given point [29].

**Figure 6:**
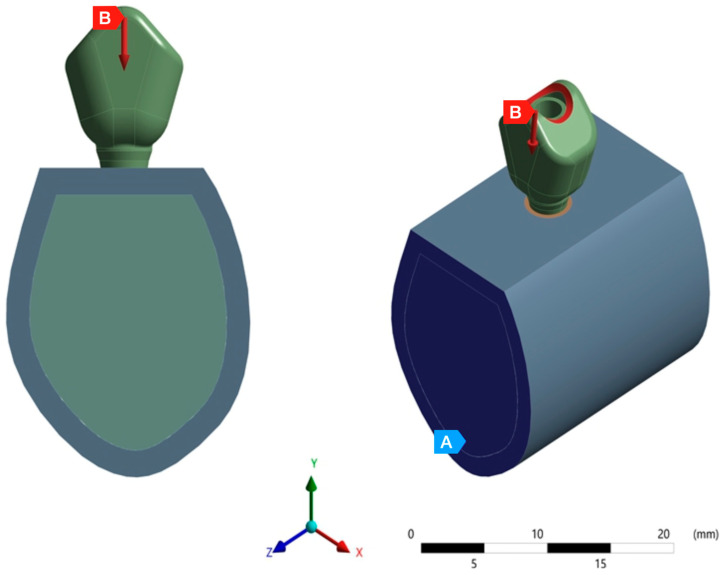
The image shows the application of 150 N of force (B) in the central fossa with an angulation of six degrees with respect to the axial axis and fixed support (A).

Additionally, the results of the observational study by Watanabe et al. [29] were used for the selection of the direction and magnitude of the applied load (150 N were applied to the central fossa of the crown at a six-degree angle with respect to the axial axis of the implant and in the direction of the lingual vestibule), simulating the load applied on a second premolar or a first molar [30,31,32]. Both stress (according to von Mises criteria) and deformation were determined numerically (Figure 6).

The models were totally constrained along their boundaries, allowing no degrees of freedom of movement.

## 3. Results

The findings showed the highest and lowest von Mises stress values, as well as the stress distribution in the bone surrounding the implant, in the implant itself, and in the prosthetic elements, as well as the deformation of all components in the three models (A, B and C).

The results obtained for minimum and maximum stress and deformation for each of the components mentioned in each of the models are presented in Table 3.

**Figure 7:**
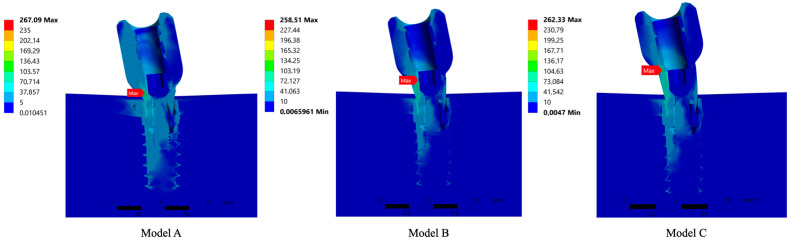
Three-dimensional study models, (Model **A**) with 1mm height abutment and 8mm height crown, (Model **B**) with 2 mm height abutment and 7 mm height crown and (Model **C**) with 3mm height abutment and 6mm height crown, with their corresponding von Mises stress location point expressed in MPa scaled to 150 MPa.

The images have been grouped by simultaneously analysing the complete assembly or the same part in the different models and studying both the stress and deformation values obtained (Figure 7).

The results showed that the longer the abutment length, the greater the stress transmitted to the bone-implant-prosthesis assembly, particularly at the bone level.

This phenomenon, which is evident in a one-dimensional lever arm system, becomes more complex when analysed in a three-dimensional assembly of parts with complex geometries, each of which also has different material properties. Therefore, considering the stress maps in Figure 4, it can be stated that the maximum stress was produced in the abutment of model C with 267.09 MPa, due to its longer lever arm, followed by the abutment of model B with 262.33 MPa and the abutment of model A with 258.51 MPa. Moreover, Figure 4 shows that the area where the highest peak stress distribution occurred for the three models was at the transition between the crown and the abutment, this being the most critical point.

Regarding the deformation of the assemblies, given the magnitude of the force, all the deformations would be practically inappreciable in images in which the deformation is at a 1:1 scale, therefore, following the usual methodology in FEA, these deformations have been magnified. The largest displacement values were found in the crown in all cases and increased with increasing abutment height. Considering that this area has a certain freedom of movement that is not impeded by any other part, it is consistent with the fact that the displacements in this area are the highest.

**Figure 8:**
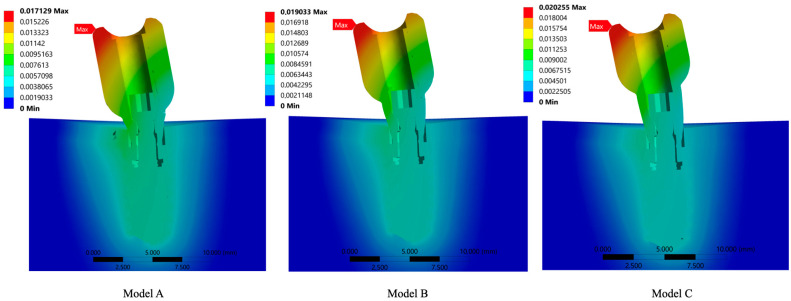
Three-dimensional study models, (Model **A**) with 1 mm height abutment and 8mm height crown, (Model **B**) with 2 mm height abutment and 7 mm height crown and (Model **C**) with 3 mm height abutment and 6mm height crown, with their corresponding deformation.

The gradual colour change observed in Figure 8 is evidence of the existence of good osseointegration in the model, which leads to a solid displacement of the assembly.

It should also be noted that in all models the deformation in the bone area was similar, being 0.0074371 for model A, 0.0074682 for model B, and 0.00749 for model C. This indicates that deformation occurred mainly in the prosthetic elements and, above all, it evidences that there was no displacement boundary between the bone tissue and the implant. In addition, it has been demonstrated that the contacts applied to the model were correct and that a behaviour known in finite element terminology as *bond* was ensured at all times, i.e., all the elements were perfectly joined together, resembling a situation of perfect osseointegration.

However, all models presented a significant increase in stresses in the most coronal region of the bone in contact with the implant. This distribution can be explained by the principle of composite beam analysis, which states that the stress transferred to the peri-implant bone is distributed mainly towards the side corresponding to the direction of the vector of the applied load. In this case, this vector had a buccolingual direction, so the stress was distributed mainly in the lingual sector of the bone surrounding the implant. Furthermore, a certain distribution of the transferred stress in the bone adjacent to the apex of the implant corresponded to the axial component of the load applied to the model.

When analysing the results focusing exclusively on the abutments, it can be observed that the deformation of the abutments was higher as their size increased, with the greatest deformation corresponding to the abutment for model C (0.017565), followed by that of model B (0.01661) and finally that of model A (0.015029). These results are consistent with the stress results observed and support the influence of the lever arm on the prosthetic system. These results can be related to the peri-implant bone loss produced by the compression of bone tissue when the prosthetic system is subjected to functional loads and there is a transmission of this force throughout the entire system, affecting each of its component elements.

The results of transferred stress and strain have to be interpreted in a way that could clinically influence implant-supported rehabilitation. In this sense, high stress transferred to the prosthetic components could lead to high deformation of the components, which could exceed the elastic limit of the components or even the fracture limit and cause mechanical and technical complications, with the most common being the chipping of the prosthetic ceramic or the fracture of the screw. If this situation of high deformations and transferred stresses are transmitted to the peri-implant bone, it is known that the bone undergoes adaptive modifications depending on the load and it undergoes deformation. This is why excessive loading and deformation would cause the bone to enter a window of overload and could lead to microfractures and bone resorption around the implant [33].

## 4. Discussion

In order to carry out this study, a FEA with the replication of abutments and implants in 3D models was used to compare the magnitude and distribution of stress and deformation of all the elements of the system, in particular the prosthetic abutment and the bone. To this end, abutments of three different heights were created, with an identical size to the entire prosthetic system. The use of FEA for biomechanical studies in oral implantology is widely accepted in the scientific literature, as shown by numerous meta-analyses and systematic reviews [34,35], and allows us to perform studies that would be more complex in vivo.

Our study has required assuming various simplifications, which have been necessary to allow the design and obtainment of results. Among them, having considered the bone as isotropic and with linear elasticity and homogeneous, having assumed complete osseointegration of the implants or a lack of total mobility between the retentive elements. However, we believe it is convenient to note that these simplifications are those commonly applied in studies of the same methodology, although with different objectives [36].

The results of this study suggest that the maximum transferred stresses in all models occur in the area with a large difference in the finite element node size between the abutment and the implant, which generates singularities when the finite element model proceeds to the application of the equations of the inter-part contacts. Based on previous evidence on finite element modelling, the maximum values determined by the model for this area may be unrealistic. Despite the typical characteristics of finite element models, the overall quality of the model and the reliability of its results are ensured by the stress distribution, represented graphically in the form of colours, observed in the bone and implant zones and at their interfaces. Therefore, in the areas where the transition of colour from bone to implant is gradual, the homogeneity of the stress distribution and its continuity are confirmed, which guarantees the reliability of the results obtained.

It should also be noted that the scale of the images generated from the stress has been limited to 150 MPa to facilitate their interpretation. The limit value to be selected must be high enough to capture the details of the metal parts, without being so high that information is lost in the bone tissue, which would mean that all the stresses would be in a range determined by similar colours and, therefore, it would be difficult to distinguish the values between different areas. These limitations with the indicated scales have been necessary due to the phenomenon of bending or the nodal concentration of singular stresses, which is very common in finite element models. However, these models provide a straightforward interpretation of the stress distribution: higher values exist in those areas of the colour map coloured with a shade different from dark blue. As noted in the article by Pérez-Pevida et al., the colourimetry of the models is key to understanding biomechanical behaviour [36].

Following the analysis of these findings, the hypothesis proposed in this study has to be rejected; considering that this is a lever arm system, the results confirm that the greater the height of the Ti-base abutment, the greater the deformation of the abutment, which leads to greater transferred stress to the implant and, therefore, greater deformation and transferred stress to the peri-implant bone. Such results should be treated with caution as they will depend to a large extent on the properties and geometries of the material, the applied load and how these conform to reality [37].

When comparing these results with those obtained in retrospective in vivo studies [3], it can be observed that the abutment height was the variable that most influenced marginal bone loss and that the prosthetic abutment with the lowest height was the one that generated the greatest bone loss. These findings offer a biological point of view, since the influence of the biological width [38] is a limitation to be taken into account when extrapolating these results. Consequently, the abutment that transmits the least load to the bone is the one that generates the greatest bone loss, since it does not respect the biological space. However, the abutment with the greatest height transfers the greatest load but maintains the biological width better.

Regarding the models developed in this research, it is assumed that all simulated structures are homogeneous, isotropic and of linear elasticity, although this does not always conform to reality, especially in bone. This is a way of simplifying the models and carrying out the analysis as validated in the literature, which includes several studies that assess the biomechanical behaviour of implant and prosthesis models assuming the same characteristics as the present study [10,36,39,40].

In addition, all three models had a cortical and trabecular bone with identical geometry and mechanical properties. This is consistent with most of the biomechanics studies on finite elements, although some studies establish a type of transitional bone in contact with the implant with a different modulus of elasticity and Poisson’s ratio than the rest of the modelled bone [41]. This study assumes that both the trabecular and cortical bone have intimate and 100% contact with the implant surface, simulating complete osseointegration of the entire surface [40,42,43].

Concerning the load applied to the models, in this study, a load of 150 N was applied to the central fossa of the modelled crown at a six-degree angle relative to the axial axis of the implant to simulate the average values that occur during functional chewing in patients with an implant-supported prosthesis. However, during the mastication process, complex loading patterns that are difficult to reproduce in this type of study occur, which implies a simplification of the loads when simulating them in the finite element models. Similarly, the functional loads caused during mastication are dynamic, but those used in the models are static, corresponding to the force’s characteristic of centric bruxism. Moreover, the type of load and the elastic properties of the modelled materials may influence the biomechanical result and should be considered as a limitation when interpreting the results. Several studies that apply similar loads can be found in the literature, which allows us to validate the loads applied in this research [44,45,46,47].

Another limitation to be considered when interpreting the results is that modelling of the soft tissues has not been carried out. The complexity of modelling soft tissue, as well as the complexity of its mechanical properties as it is a non-rigid element, makes its application in finite element studies challenging. Other studies on abutments performed on patients, such as that by Galindo-Moreno et al. [3], reported the importance of soft tissues and, when interpreting the results, tissular biology, the importance it has on the prosthetic system and the fact that the peri-implant bone should be considered. In this sense, extrapolating the mechanical results and applying the concepts of tissular biology, opposing results are found, since from a mechanical perspective, the longer abutments transfer greater tension and, therefore, the bone-implant system suffers greater deformation. However, these abutments maintain larger tissue volumes and have been shown to better maintain the peri-implant bone compared to shorter abutments with less peri-implant soft tissue [48,49]. In this regard, as Frost’s mechanostat theory proposes, bone adaptation to load transfer, translated into deformation, can be influenced by both an excess and a defect of load and therefore the mechanical results obtained are hardly comparable with the biological results, even if they are opposed to each other.

In this sense, it should be considered that the magnitude of deformation and load transferred to the peri-implant bone determined in the test are within acceptable ranges with a physiological and adaptive bone response and, therefore, would not pose a risk to the osseointegration of the implant in any situation.

In addition, the choice of the restorative material for crowns on abutments has been based on the evolution of CAD-CAM design prostheses and, therefore, the most commonly used material is milled zirconia. This is consistent with studies that evaluated the effect of the use of different prosthetic materials on implant stress distribution and peripheral bone structure and concluded that the choice of prosthetic material is not determined and has only a minor effect on stress patterns [47,50,51]. In this study, the crowns show similar behaviour, even though the crown size is reduced as the abutment size increases to keep the size of the prosthetic system constant.

Finally, another limitation to be taken into account is the screw preload. Jorn et al. [52] indicate that the screw preload should be included in a dental implant investigation for a realistic study of the implant complex. Nevertheless, in the present study, the screw preload is omitted to simplify the system and only the stress applied and its deformation are analysed, considering it as one more element of the assembly and with an intimate connection of 100%. This simplification is found in most of the finite element tests described in the literature, which evaluate the behaviour of implant-supported restorations [46,53,54]. However, the results show that as the abutment size increases, the stress and deformation suffered by the prosthetic screw increases, which may favour screw loosening and in extreme cases, extrapolating the results to clinical practice, may lead to fatigue fracture.

## 5. Conclusions

Considering the limitations inherent to this type of analysis and the simplifications assumed, it can be concluded that the stress transferred to the bone-implant system is determined by the height of the abutment used in the implant-supported prosthesis, with the transferred stress increasing as the abutment height increases. Moreover, as the abutment height increases, a greater deformation of all the elements of the prosthesis-implant-bone system occurs, all of them being within the physiological ranges. Proper treatment planning prior to surgery to determine the position of the implant and the size of the prosthetic abutment to be used is of great importance; priority should be given to the use of abutments of an intermediate height that respect both the biological width and acceptable ranges of transferred stress. The use of any of the evaluated abutments is within the proper biomechanical range consistent with the biological results found in the literature. Therefore, a greater increase in abutment height would be ideal for the maintenance of the peri-implant marginal bone, combined with proper biological and biomechanical behaviour, assuming that the longer the abutment, the greater the load transferred and the greater the deformation. It would be of great interest to develop similar studies in vivo to contrast the results obtained, as well as to promote the development of abutments with different macroscopic designs to reduce the load transferred to the peri-implant bone.

## Figures and Tables

**Table 1:** Elastic characteristics of the modelled components.

MATERIAL	Component	Young’s Modulus (GPa)	Possion’s Ratio
Cortical Bone		1.5 × 10^10^	0.30
Trabecular Bone		1 × 10^9^	0.25
Grade 4 Titanium	Implant	1.07 × 10^11^	0.35
	Abutment	1.07 × 10^11^	0.35
	Screw	1.07 × 10^11^	0.35
ZrO_2_	Crown Structure	2.1 × 10^11^	0.32

**Table 2:** Components nodes and elements.

Components		Abutment A	Abutment B	Abutment C
Screw	Nodes	1098	1098	1098
	Elements	514	514	514
Abutment	Nodes	20,627	21,713	15,478
	Elements	12,169	12,918	9250
Implant	Nodes	101,939	101,923	101,931
	Elements	65,606	65,606	65,614
Trabecular Bone	Nodes	82,354	82,354	82,063
	Elements	51,310	51,310	51,099
Cortical Bone	Nodes	8675	8675	8656
	Elements	4634	4634	4609
Crown	Nodes	6693	6711	6711
	Elements	3939	3980	3980

**Table 3:** Results obtained in terms of stress and deformation in the three models.

	ABUTMENT A		ABUTMENT B		ABUTMENT C	
Element	Von Misses Stress (MPa)	Microstrains	Von Misses Stress (MPa)	Microstrains	Von Misses Stress (MPa)	Microstrains
System	0.0065961–258.51	0–0.017129	0.0047–262.33	0–0.019033	0.010451–267.09	0–0.020255
Crown	0.42295–175.16	0.0049136–0.017129	0.43485–194.19	0.0056471–0.019033	0.43871–194.52	0.0067211–0.020255
Abutment	0.48626–258.51	0.0052729–0.015029	0.61873–262.33	0.0056696–0.01661	0.9399–267.09	0.0053955–0.017565
Screw	0.010451–35.017	0.0059213–0.0080568	0.006596–36.467	0.0059142–0.008087	0.0047–40.64	0.0059164–0.008274
Implant	0.068522–98.854	0.0053687–0.007732	0.10273–99.97	0.0053543–0.007735	0.10294–109.44	0.0053586–0.008014
Cortical Bone	0.0066727–32.344	0–0.007468	0.0077927–33.851	0–0.007677	0.011497–45.608	0–0.0081
Trabecular Bone	0.0066727–25.158	0–0.0072328	0.0077927–30.3165	0–0.007266	0.011457–35.475	0–0.0081

## Data Availability

The original contributions presented in the study are included in the article, further inquiries can be directed to the corresponding author.
